# Younger age as a prognostic indicator in breast cancer: A cohort study

**DOI:** 10.1186/1471-2407-11-383

**Published:** 2011-08-28

**Authors:** Elrasheid A H Kheirelseid, Jennifer ME Boggs, Catherine Curran, Ronan W Glynn, Cara Dooley, Karl J Sweeney, Michael J Kerin

**Affiliations:** 1 Department of Surgery, National University of Ireland, Galway, Ireland; 2 Biostatistics Unit, National University of Ireland, Galway, Ireland

## Abstract

**Background:**

The debate continues as to whether younger women who present with breast cancer have a more aggressive form of disease and a worse prognosis. The objectives of this study were to determine the incidence of breast cancer in women under 40 years old and to analyse the clinicopathological characteristics and outcome compared to an older patient cohort.

**Methods:**

Data was acquired from a review of charts and the prospectively reviewed GUH Department of Surgery database. Included in the study were 276 women diagnosed with breast cancer under the age of forty and 2869 women over forty. For survival analysis each women less than 40 was matched with two women over forty for both disease stage and grade.

**Results:**

The proportion of women diagnosed with breast cancer under the age of forty in our cohort was 8.8%. In comparison to their older counterparts, those under forty had a higher tumour grade (p = 0.044) and stage (p = 0.046), a lower incidence of lobular tumours (p < 0.001), higher estrogen receptor negativity (p < 0.001) and higher *HER2 *over-expression (p = 0.002); there was no statistical difference as regards tumour size (p = 0.477). There was no significant difference in overall survival (OS) for both groups; and factors like tumour size (p = 0.026), invasion (p = 0.026) and histological type (p = 0.027), PR (p = 0.031) and *HER2 *(p = 0.002) status and treatment received were independent predictors of OS

**Conclusion:**

Breast cancer in younger women has distinct histopathological characteristics; however, this does not result in a reduced survival in this population.

## Background

Breast cancer accounts for approximately 23% of all female malignancies, and its incidence is increasing, especially in the developed countries [1]. In young women, the incidence of the disease is low (< 17 cases per 100,000 women or < 6% of all breast cancers among women of any age) [2,3]; however, accumulating evidence suggests that breast cancer in this age group is more aggressive and associated with poor outcome than in their older counterparts [4-6]

Although some reports have identified young age at diagnosis as an adverse prognostic indicator [7], this could be ascribed to a combination of factors, including delayed presentation, advanced disease stage and unfavorable tumour characteristics [8,9]. Furthermore, the annual risk of recurrence appears to be constant throughout life, therefore the younger the age at diagnosis, the higher the accumulated lifetime risk of recurrence [10].

The objectives of this study were to determine the incidence of primary operable breast cancer in women under 40 years old and to analyse the clinicopathological characteristics and outcomes in this group of patients compared to those over forty.

## Methods

### Patient cohort

Data was acquired and collated from the prospectively reviewed Galway University hospital, Department of Surgery breast cancer database from 1989 through to 2009. Ethical approval for this database, and therefore any study that could arise from it, was granted by the Clinical Research Ethics Committee, Galway University Hospitals and written consent was obtained from each of the patients before have their clinical data was recorded and then updated. The resulting data was further queried for cases under the age of 40 years. All patients were treated according to local protocols and followed three monthly for one year, 6 monthly for two years and then annually. Male patients and women who developed bilateral breast tumour were excluded from the study.

Data collected included patient demographics and year of presentation, tumour size, grade, stage, histological type, extent of tumour invasion and lymph node involvement and the presence or absence of local or distant metastasis. Estrogen (ER), progesterone (PR) and *HER-*2/*neu *receptor status was also noted where data was available, as was local therapy and the use of hormonal therapy, chemotherapy and radiotherapy.

Prior to the advent of Tamoxifen use in the late 1980s, bilateral prophylactic oopherectomy was performed routinely in premenopausal women with breast cancer. We used this group of patients to evaluate the impact of ovarian ablation on survival of young women with breast cancer.

The SPSS^® ^17.0 software package (SPSS Inc., Chicago, IL, USA) was used for statistical analysis. Mann-Whitney U and t-tests were used, as appropriate, for comparison of continuous variables and the Chi-square test was employed for analysis of categorical variables. All tests were two sided and a result was considered significant if the calculated *P *value was < 0.05.

For survival analysis, each woman less than 40 was matched to the two closest controls (women over forty) for both disease stage and grade using a Malanhobis distance based on ranks, and the matching was carried out using the Optmatch package [11,12]. Survival distributions were analyzed using the Kaplan-Meier method. The statistical significance of differences in survival between groups was determined by log rank. Multivariate analysis was done using Cox regression, while logistic regression was employed for categorical data.

## Results

There were 2869 cases of breast cancer identified in the 20-year study period. The number of women diagnosed with breast cancer under the age of forty in the database was 276 (8.8%) (Table 1). No significant difference in the size of the tumour at time of diagnosis was noted between the two groups (p = 0.477). Younger women were more likely to present with grade 3 disease (36.7% *vs*. 29.3, *p = 0.044*) and women older than 40 years of age were more frequently diagnosed with invasive lobular carcinoma than younger women (15.2% *vs*. 7.55, *p < 0.001*). Although the most common presenting stages were similar in both groups, women less than 40 years of age were more likely to present at stage II, whereas women older than 40 years were more likely to present at an earlier stage (*p = 0.046*).

**Table 1:** Characteristics of breast cancer in less than 40 yrs women compared to more than 40 yrs group

Variable	Less than 40 (n = 276)	More than 40 (N = 2869)	*P Value*
**Age in yrs (SD) **^#^			< 0.001*
Mean	35.81 (3.92)	60.08 (12.22)	
Median (range)	37 (20-40)	59 (41-96)	

**Tumour size (mm) **^#^			0.477
Mean	21.49	22.74	
Median (range)	20 (1-120)	20 (1-160)	

**Tumour grade**			0.044*
Grade 0	69 (27%)	697 (26.47%)	
Grade 1	23 (9%)	262 (9.9%)	
Grade 2	70 (27.3%)	912 (34.5%)	
Grade 3	94 (36.7%)	774 (29.3%)	

**Histology Type**			< 0.001*
Ductal	178 (74.2%)	1768 (72.8%)	
Lobular	18 (7.5%)	368 (15.2%)	
Others	44 (18.3%)	291(12%)	

**Tumour stage**			0.046*
0	17 (7.4%)	210 (9.2%)	
I	66 (28.6%)	538(23.6%)	
II	93 (40.3%)	850 (37.3%)	
III	46 (19.9%)	477 (20.9%)	
IV	9 (3.9%)	202 (8.9%)	

**ER status**			< 0.001*
Positive	123 (58.3%)	1629 (74.5%)	
Negative	88 (41.7%)	559 (25.5%)	

**PR status**			0.319
Positive	102 (70.3%)	1142 (72.5%)	
Negative	43 (29.7%)	433 (27.5%)	

**Her-2 status**			0.002*
Positive	34 (30.6%)	213 (18.6%)	
Negative	77 (69.4%)	935 (81.4%)	

**Surgical treatment**			< 0.001*
Mastectomy	192 (75.9%)	1647 (61.9%)	
BCS	61 (24.1%)	1012 (38.1%)	

**Adjuvant hormonal therapy**			0.005*
Yes	184 (86.4%)	2924 (92.4%)	
No	29 (13.6%)	159(7.6%)	

**Adjuvant chemotherapy**			0.021*
Yes	109 (46.2%)	866 (38.4%)	
No	127 (53.8%)	1389 (61.6%)	

**Adjuvant radiotherapy**			0.778
Yes	135 (59.2%)	1234 (58%)	
No	93 (40.8%)	892 (42%)	

^# ^Mann-Whitney u test, ER = Estrogens receptor, PR = Progesterone receptor

### Tumour biology

There were significant differences in ER status (p < 0.001) and *HER2*/*neu *status (p = 0.002). Approximately 42% of tumours in women less than 40 years of age were ER negative and 30.6% were *HER2*/*neu *positive; this compared with 25.5% and 18.6% in women over 40, respectively. There were no differences according to PR status (p = 0.319).

### Management of breast cancer in young women in our institution

Excepting adjuvant radiotherapy, a significant trend towards more aggressive management was noted in the younger women cohort. They received both mastectomy (75.9% *vs*. 61.9%, *p < 0.001*) and adjuvant chemotherapy (46.2% *vs*. 38.4%, *p = 0.021*) more frequently than older women independent of disease stage. However; the difference in adjuvant chemotherapy noted between the two groups might be due to the large number of women > 60 years in age (only 302 of the 1278 women over 60 years old received chemotherapy). On the other hand, women older than 40 years more frequently receive hormonal therapy (92.4 *vs*.86.4%, *p = 0.005*). About 159 women in the more than 40 years old group (7.6%) opted out of the hormonal therapy due to intolerable side effects.

### Survival analysis

Each of the younger breast cancer patients (n = 276) was matched for stage and grade to two older control patients (n = 552). Moreover, all the patients involved in the study were of the same ethnic origin. Median (interquartile range) follow-up was 194 (128-260) months for both groups.

**Figure 1:**
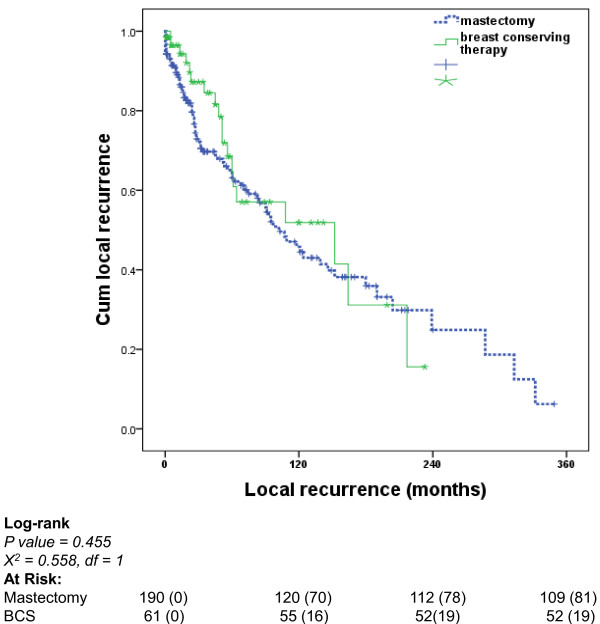
**Local disease recurrence in women less than 40 who were treated by mastectomy compared to breast conserving surgery (BCS)**.

No significant difference in disease free survival (DFS) was identified comparing the two groups using multivariate Cox regression analysis (p = 0.150). Factors like nodal status (p = 0.012), adjuvant hormonal therapy (p = 0.047) and adjuvant chemotherapy (p = 0.015) emerged as independent predictors of disease recurrence. Furthermore, we compared the local recurrence rates (LR) in less than 40 years group based on the surgical treatment they received. No significant difference in LR was determined comparing those who underwent mastectomy to those who had breast conserving surgery (BCS) using both univariate and multivariate analysis (Figure 1). The median local recurrence-free survival for women underwent mastectomy was 102 (76-127) months compared to 152 (19-284) months in the BCS group

**Figure 2:**
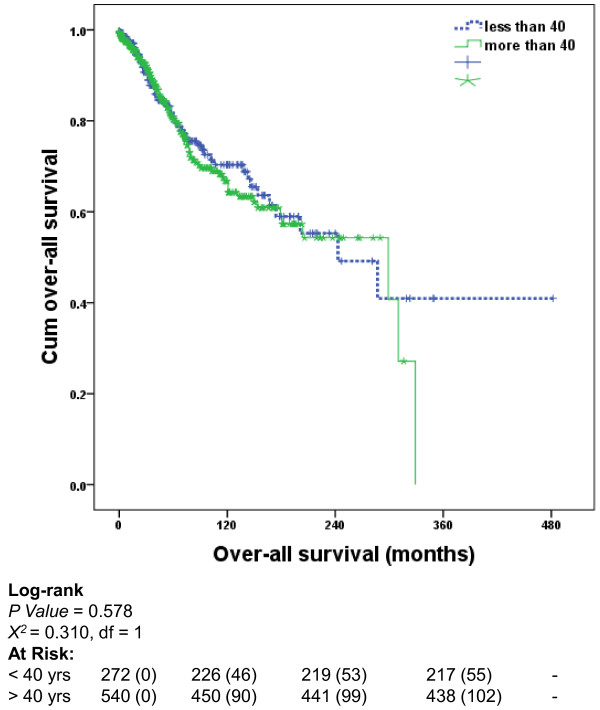
**Overall-survival analysis in women less than 40 compared to their older controls**.

Overall survival was calculated as the number of months from the diagnosis of the tumour to death or last follow-up. The median survival for women less than 40 years of age was 243 (88-300) months while that for the older cohort was 299 (75-329) months. No significant difference in overall survival (OS) was noted when comparing the two groups (Figure 2). On Cox regression analysis, tumour size (p = 0.048), stage (p = 0.018) and PR status (p = 0.020) were independent predictors of survival. After adjustment for these variables, the OS in both groups did not differ significantly (p = 0.587) (table 2).

**Table 2:** Cox proportional hazards model of survival in all patients

Variable	B	SE	Wald	df	**Sig**.	Exp(B)
**Age at diagnosis**	.019	.012	2.661	1	.103	1.019

**Category**	-.566	.355	2.539	1	.111	.568

**Tumour size**	.022	.007	8.981	1	.003*	1.022

**Tumour grade**	.097	.095	1.027	1	.311	1.101

**Histological type**	-.062	.105	.353	1	.552	.939

**Tumour invasion**	.009	.159	.003	1	.956	1.009

**Nodal status**	.131	.089	2.165	1	.141	1.140

**Distant metastasis**	.107	.272	.154	1	.695	1.113

**ER receptors status**	.039	.036	1.161	1	.281	1.040

**PR receptors status**	.119	.051	5.351	1	.021*	1.126

**HER2 status**	-.090	.056	2.591	1	.107	.914

**Hormonal therapy**	-.264	.389	.462	1	.497	.768

**Chemotherapy**	-.007	.079	.008	1	.927	.993

**Radiotherapy**	-.003	.010	.071	1	.790	.997

**Surgical treatment**	.011	.209	.003	1	.957	1.011

The output of this analysis includes the unstandardized regression coefficient (B), the standard error of B and its Wald test significance value, the degrees of freedom and the significance value of the coefficient. If the significance value for the coefficient is more than 0 050, then the co-variate effect cannot be assumed to be different from 0. The predicted change in the hazard per unit increase in the co-variate is Exp (B); if the value is less than 1, then the direction of the effect is towards reducing the hazard rate. To be considered to have a significant effect on the hazard rate, the 95 per cent confidence interval for the Exp (B) should not include 1.

Category = less than 40 yrs group *vs*. more than 40 yrs group

(*) = significant values.

df = degree of freedom

B = unstandardized regression coefficient;

Exp (B) = predicted change in the hazard per unit increase in the co-variate

In order to address the effect of bilateral prophylactic oopherectomy, we compared the overall survival for women less than 40 years of age who underwent oopherectomy, to that in those who did not. The majority of patients who were offered oopherectomy were stage I and II disease (86%), and 76% of them had mastectomy as surgical treatment modality. As adjuvant treatment 100% in oopherectomy group received hormonal therapy, 36% had radiation therapy and 24% had chemotherapy. No significant difference in survival between the two groups was determined using both univariate and multivariate analysis. Of further interest, the group of patients who did not receive oopherectomy lived longer than those who had bilateral oopherectomy (mean survival of 288 months vs. 264 months (*log-rank p = 0.215, X^2 ^= 1.54*).

## Discussion

Breast cancer in patients under 40 years of age is uncommon; however, it has generated considerable interest because of the associated unfavorable outcome reported in several studies [6,7,13,14]. Younger age has been generally accepted as an independent adverse prognostic indicator of survival in breast cancer [15-18]. Nevertheless, many reports suggest that the poor outcomes associated with this age group are complicated by several additional factors [5,13,19]. Given the lack of routine screening programmes for women younger than 40 years, it is not surprising that women in this age group are more likely to present with a palpable mass and that their tumours tends to be larger and are more likely to have nodal involvement, than tumours detected by screening [5,13,20].

It has been determined that underlying tumour pathology such as higher tumour grade, nodal status and presence of distant metastasis at diagnosis contribute to the worse outcome in breast cancer in women less than 40 years of age [4,5,21,22]. In relation to receptor status, tumours in young women have been predominantly reported as ER and PR negative, and have also been shown to over-express Her2/neu [6,13,19,23,24]. In addition; the rates of the known aggressive triple negative (PR, ER and HER2 -ve) tumour, which carries high risk of recurrence, were reported to be higher in young females [25]. In this study, younger women had tumours that were distinctly different from those in older women and were characterized by previously identified unfavorable biological parameters. Histopathological analysis showed that the majority of younger women were diagnosed with high grade and advanced stage tumours. Invasive ductal carcinoma was common in both groups. Although rare in our cohort of patients, invasive lobular carcinoma was more common in older women, a finding similar to that published in the literature [26,27]. Furthermore, biological evaluation of breast cancer in young women group revealed higher frequency of ER negativity and *HER2/neu *overexpression. No significant difference was identified in PR status, although it should be noted that the majority of patients in both groups were PR positive (70%). These findings strongly support accumulating evidence that breast cancer in young patients is biologically more aggressive and associated with unfavorable prognostic markers relative to their older counterparts.

The accumulating evidence of biologically unfavorable breast cancer among younger women has resulted in more aggressive treatment strategies for this patient population. Hence, there is a very low threshold towards more aggressive surgical treatment of breast cancer in young females. Although BCS has been found in some studies to be associated with higher rates of local recurrence after long term follow-up [28,29], numerous studies have failed to confirm the superiority of mastectomy over breast conserving surgery (BCS) in improving both the disease-free and overall survival. Of those is a recent report by Livi et al., who analysed the breast cancer outcome in 346 young females and found no significant role of surgical treatment (mastectomy *vs*. BCS) as predictor of local recurrence [22]. In this study, and in keeping with literature, although the majority of our young patients underwent mastectomy, we found no significant difference in local recurrence-free survival between the mastectomy and BCS groups.

The role of postoperative radiotherapy in reducing breast cancer local recurrence has been confirmed in many studies [30-34]. However; there is conflicting evidence in translating its role in controlling local recurrence into breast cancer mortality reduction [30,33,35,36]. Significant survival improvements have been reported in subgroup analyses of patients with favorable prognostic indicators like: grade I disease, less than 3 positive lymph nodes, tumours less than 2 cm and hormone receptors positive disease, but not in high risk groups [33,35,37-39]. Adjuvant chemotherapy has been shown to benefit young patients with approximately 37% reduction in recurrence rates and a 27% reduction in death rates [40,41]. With both single-agent chemotherapy and polychemotherapy, there is trend towards greater benefits among younger women, but both for recurrence and for mortality the age-standardized effect of single-agent regimens were significantly less favorable than those of the polychemotherapy regimens [42]. Regarding chemotherapeutic agents, it has been widely accepted that anthracyclines and taxanes are the most effective agents in adjuvant settings of breast cancer management in young females. A meta-analysis by the early breast cancer trialists' collaboration group (EBCTCG) showed that 6 months of anthracycline-based polychemotherapy reduces the annual breast cancer death by about 38% for women younger that 50 years of age at diagnosis [42].

Hormonal therapy effect had been considered of secondary importance in young females with breast cancer. However; it has become common current practice to follow adjuvant chemotherapy in receptor positive young women. This practice is based on the evidences provided by recent trials [42-46]. Hormonal therapy value in premenopausal women has been defined recently in a meta-analysis of randamised trials which showed that combination of chemotherapy with 5 years Tamoxifen in ER positive breast cancer reduce risk of death by 57% [42]. In addition, the intermediate results of the international breast cancer study group trial confirmed that Tamoxifen significantly improve outcome in premenopausal women with hormone receptors positive disease [44].

Therefore, in our cohort of patients, those less than 40 years of age were managed aggressively independent of the disease stage. Further elucidation of tumour characteristics such as HER2neu status has given adjuvant therapists the opportunity to tailor systemic treatment and clearly the younger patients are more suited to this treatment based on our and others findings

Previous reports comparing survival between women less than and greater than 40, with breast cancer have returned inconsistent results. Both Kollias et al. and Yildirim et al. found decreased DFS and OS in younger patients [17,18]. However; other studies have demonstrated that, young age in its own is not an adverse prognostic factor [13,47]. The small number of patients, study design and the employment of different statistical methods might explain these variations. Moreover, only a few studies have assessed the prognostic indicators in order to confirm whether the poorer outcome in younger women could be related to the biological characteristics of their tumours. By controlling for confounders and employing multivariate analysis methods, we have been unable to identify significant difference in both DFS and OS between the two groups of patients. Consistent with McAree et al [19], we identified PR receptor positivity as a predictor of survival in younger women with breast cancer. In additions; other variables like tumour size and stage were shown to influence OS while nodal status and adjuvant chemoendocrine therapy predicted DFS in women less than 40 by multivariate analysis. We have failed to identify any benefit of ovarian ablation in improving OS in younger women. Interestingly, the group of patients who received bilateral prophylactic oopherectomy had poorer OS than those who did not. This finding could be explained by the fact that most of our patients had adjuvant chemo-endocrine therapy. EBCTCG overviews have shown that, although ovarian ablation is associated with improved survival for premenopausal women both on recurrence and on breast cancer mortality, the effect of ovarian treatment appear to be smaller in the trials where both groups got chemotherapy [42]. In addition, they reported that more breast cancer related deaths were noted in the trials of ovarian ablation in the presence of chemotherapy than in the trials of ablation in the absence of chemotherapy [48-50]. This could partially be explained by the toxic effect of chemotherapy on the ovary, limiting the benefits of other ovarian treatments.

## Conclusions

This study has demonstrated that women less than 40 years of age present with higher grade and poorly differentiated tumours. Moreover; younger women had tumours that were more likely to be ER negative and *HER2/neu *receptor over-expressed. However; we have shown no difference in both disease-free survival and over-all survival between this group of patients and women over 40 years of age.

## Competing interests

The authors declare that they have no competing interests.

## Authors' contributions

EAHK designed the study, was responsible for data analyses and drafted the manuscript. JMEB and CC contributed to data collation. RWG contributed to manuscript editing. CD contributed to statistical analysis of the survival data. KJS and MJK conceived and designed the study, critically reviewed the manuscript and participated clinically. All authors read and approved the final manuscript.

## Pre-publication history

The pre-publication history for this paper can be accessed here:

http://www.biomedcentral.com/1471-2407/11/383/prepub
